# Exploring Patient and Podiatrist Perspectives of the ‘In‐Remission’ Status in Diabetes‐Related Foot Disease

**DOI:** 10.1002/jfa2.70045

**Published:** 2025-05-13

**Authors:** Gordon Donaldson, Gordon Hendry, Ruth Barn

**Affiliations:** ^1^ Department of Podiatry and Radiography School of Health and Life Sciences Glasgow Caledonian University Glasgow UK

**Keywords:** diabetes foot ulcer prevention, diabetes foot ulcer recurrence, patient education, risk stratification

## Abstract

**Background:**

The term ‘in‐remission’ has been historically associated with the disease status in cancer. In diabetes foot care, the term was introduced into risk stratification systems in order to support patient communication and healthcare prevention strategies post‐ulceration. However, despite the inclusion of an ‘in‐remission’ category into risk stratification systems, the adoption and perception of the terminology in clinical practice remains unknown. The aim of this study is to explore patient and clinician perceptions of the term ‘in‐remission’ in the context of diabetes foot disease management.

**Methods:**

Semi‐structured focus groups or interviews were conducted to identify the perceptions and impact of the term ‘in‐remission’ on end users. Participants included patients classified as ‘in‐remission’ as well as podiatrists who routinely work with people who have diabetes. Recruitment occurred via social media adverts, posters and snowball sampling. All focus groups and interviews were recorded and transcribed verbatim. Data were analysed for themes.

**Results:**

The study included *n* = 9 people with diabetes classified as in‐remission and *n* = 12 podiatrists. One online focus group was held with *n* = 5 podiatrists, whereas individual telephone interviews were conducted with the remaining *n* = 7 podiatrists and all *n* = 9 participants with diabetes. Three overarching themes were identified with several sub‐themes in each group. Perceptions and implementation of the term ‘in‐remission’ were highly variable whereby podiatrists tend to avoid using the term directly with patients and instead focus on patient education and held mixed views of the impact on patients. People with diabetes were largely unaware of their ‘in‐remission’ status and had varied opinions on what ‘in‐remission’ means and how it may impact on foot care.

**Conclusion:**

Awareness and implementation of the term ‘in‐remission’ into podiatry practice are inconsistent. Despite the intention of the term to increase patient awareness and access to services, themes were identified regarding applicability and suitability resulting in avoidance by clinicians and confusion and fear from people with diabetes. These findings suggest that the term is not having the intended effect and further work is required to more fully explore the adoption of this terminology.

## 1 Introduction

The worldwide prevalence of diabetes‐related foot ulcer (DFU) is reported at 6.3%, albeit with widespread regional variation ranging from 1.5% in Australia to 13% in North America [1]. It is recognised that recurrence of DFU is highly likely with 40% of those with a healed ulcer experiencing DFU recurrence within 1 year and up to 90% within 10 years [2]. Not only are DFU a leading cause of the global burden of disability [3] but they also comprise a substantial portion of the overall cost of diabetes to the healthcare system [4]. Hence, proactive preventative strategies are required. Moreover, alongside amputation, they have been reported as independent risk factors for early death [5] with the 5‐year mortality rate of DFU similar to many types of cancer [6, 7]. As a result, terminology typically associated with cancer disease status, ‘in‐remission’, was proposed as a method of emphasising the comparable risk and recurrence of DFUs [8].

**FIGURE 1:**
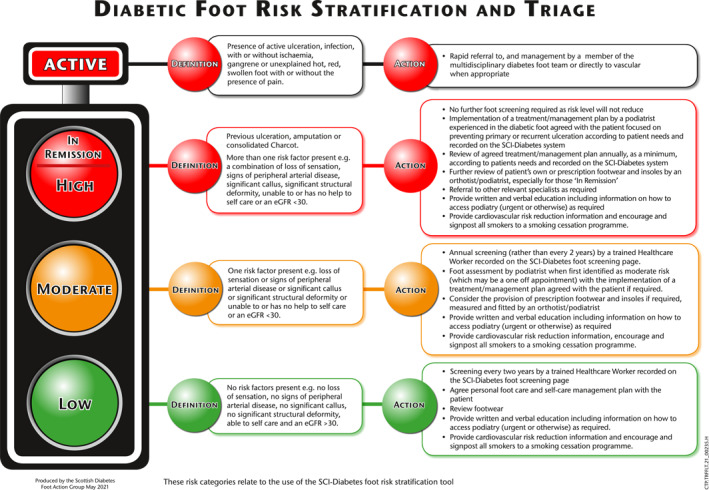
Scottish Diabetes Foot Action Group for diabetic foot risk stratification and triage [4].

The term ‘in‐remission’ can be traced back to the 13^th^ century, defined as a release from obligation and is used in oncology to mean decreased cancer signs and symptoms [9, 10]. Armstrong and Mills (2013) suggested that describing a patient with diabetes as ‘in‐remission’ rather than ‘healed’ following a period of active ulceration would highlight the temporary absence of this complication, the risk of recurrence and the need for frequent follow‐up and timely intervention. This was adopted by the Scottish Diabetes Foot Action Group (SDFAG) who, in 2016, added the ‘in‐remission’ category to their existing diabetic foot risk stratification and triage system (Figure 1) [11]. This terminology has also been endorsed by the Royal College of Podiatry [12], Diabetes UK [13] and ‘foot in remission’ was included in the International Working Group on the Diabetic Foot definitions update in 2019 [14]. Previously, patients with a history of ulceration, amputation, Charcot neuro‐osteoarthropathy or more than one risk factor for the development of these pathologies were all considered to be ‘high‐risk’ [15]. According to the revised system, they are considered to be ‘in‐remission’, whereas ‘high risk’ patients are defined as those who have not experienced these complications but are at greater risk of developing them [15].

Educational patient information leaflets aligned to the revised foot risk stratification system, including one specifically designed for people in the ‘in‐remission’ category have also been developed [16]. The term ‘in‐remission’ was specifically included in an attempt to raise awareness among patients of the risk of recurrence [16]. Leaflets were reviewed by nationally recognised bodies and endorsed by several organisations spanning professional and national boundaries [16]. However, it is not clear whether these changes have achieved their intended impact. We do not know whether taking a medical term from one population (cancer) and applying that to evoke behaviour change in another population is effective, particularly if end users are unaware of related publications, intended meaning and wider context. Moreover, lay comprehension of cancer terminology (including remission) has been reported as poor in samples from the United Kingdom [17] and the Netherlands [18]. This study aimed to explore patient and clinician perceptions of the term ‘in‐remission’ in the context of diabetes foot disease.

## 2 Methods

### 2.1 Study Design

Methods and results are reported in line with the consolidated criteria for reporting qualitative research (COREQ) 32 item checklist (Supporting Information S1: Appendix 1) [19]. This was a qualitative study design employing telephone and video conferencing based on semi‐structured interviews and focus groups. Data were obtained and analysed according to the principles of thematic analysis.

### 2.2 Recruitment

Following the receipt of ethical approval from Glasgow Caledonian University School of Health and Life Sciences Research Ethics Committee (AHP/A23/026 on 14th June 2024), participants were recruited via a combination of posters on public noticeboards, targeted social media posts on Twitter, Facebook and online diabetes support groups and forums, emails to contacts in professional networks and a snowball sampling (“word‐of‐mouth”) approach. Participant inclusion and exclusion criteria are outlined in Table 1. All potential participants who expressed an interest in the study were provided with the study information and screened for eligibility. Written informed consent was obtained prior to participation.

**TABLE 1:** Participant inclusion and exclusion criteria.

People with diabetes inclusion	People with diabetes exclusion
Age 18 or older	Classed as low–high risk
Confirmed diagnosis of diabetes	Unable to communicate in English
At least one episode of resolved diabetes foot disease (in‐remission)	Unable to participate in telephone or video call

**Clinician inclusion**	**Clinician exclusion**
Qualified podiatrist	Healthcare professional not involved in diabetes foot care
Experience working with people with diabetes and diabetes‐related foot disease	

### 2.3 Data Collection

Topic guides for patients and clinicians (Supporting Information S1: Appendix 2) were developed and used as a framework for the conversations with supplementary questioning and additional prompts where necessary. Open‐ended questions were used to mitigate potential response bias. A pilot interview and a focus group were conducted to facilitate testing of the questions, prompts, explanations and interviewing technique, with minor adaptations made to the topic guides and interview style as a result. All interviews and focus groups were conducted by the first author (GD, MSc), a final year podiatry student with no prior interaction with the participants. All data were collected between the second of July and the second of August 2024 via telephone call or Microsoft Teams dependent on individual preferences and availability. The focus group and all interviews were audio‐recorded and transcribed verbatim. Transcripts were returned to participants for verification to ensure accuracy of data.

### 2.4 Data Analysis

Transcripts were read and re‐read in full multiple times to ensure familiarity with the content (GD). An open‐coding process was conducted for each transcript whereby transcripts were reviewed and coded in isolation to ensure that codes created were representative of individual perspectives. All coded text was subsequently collated, expanded and refined over the course of the data analysis period. Overarching themes were identified from the coded data for both the diabetes and clinician participant groups. Themes were discussed by the study team at regular intervals. Four transcripts (two from each group) were independently reviewed by two members of the study team (RB and GJH) to ensure agreement with the proposed themes and determine any themes which had not yet been identified. A consensus meeting was held where team members' differing perspectives were considered and overall agreement on the final themes was reached.

## 3 Results

Eleven people with diabetes expressed an interest in participating; *n* = 2 did not meet the inclusion criteria leaving *n* = 9 people with diabetes classed as ‘in‐remission’ who took part (*n* = 2 with type 1 and *n* = 7 with type 2 diabetes). Five people reported a single ulceration which had resolved and *n* = 4 had experienced multiple ulcerations. Of the *n* = 4 participants with multiple ulcerations, *n* = 2 also had consolidated Charcot. All people with diabetes who took part in this study participated in individual interviews either by telephone or online video call. Interview duration ranged from 12 to 30 min.

Twelve podiatrists involved in the care of people with diabetes participated in the study, of which *n* = 10 were employed by NHS Scotland, *n* = 1 by NHS England and *n* = 1 by the Health Service Executive of the Republic of Ireland. Proportion of clinical time spent working with people with diabetes ranged from 10% to 100% and clinical experience ranged from 2 to 27 years (mean = 12.33 and standard deviation = 8.43). Five podiatrists participated in an online focus group and *n* = 7 podiatrists participated in individual interviews either by telephone or online video call. Interview duration ranged from 14 to 40 min. The focus group duration was 58 min.

### 3.1 Overview of Themes

#### 3.1.1 People With Diabetes

Qualitative analysis revealed three overarching themes and several subthemes from the ‘in‐remission’ participants summarised in Table 2.

**TABLE 2:** In‐remission participant themes and sub‐themes.

Themes	Sub‐themes
1. Lack of awareness of ‘in‐remission’ risk category	1.1 People with diabetes have not been informed of their ‘in‐remission’ risk status by clinicians
	1.2 People with diabetes have not accessed information leaflets
2. Varied perceptions of the meaning of ‘in‐remission’	2.1 Most people associate the term with cancer
	2.2 The association of the term with cancer gives the sense that recurrence is inevitable
	2.3 Many people with diabetes held the belief that they will one day be considered ‘cured’
3. Impact of being classed ‘in‐remission’ highly variable	3.1 Most people with diabetes felt that being classed as ‘in‐remission’ would hypothetically increase engagement with foot care
	3.2 Many people with diabetes believe that being classed as ‘in‐remission’ has a negative psychological effect and instilled a sense of fear.

Theme 1 Lack of awareness of the in‐remission risk category.

People with diabetes and a history of foot disease were generally unaware that they are considered to be ‘in‐remission’. Sub‐themes associated with this lack of awareness include not being informed of the ‘in‐remission’ risk status by clinicians and not having access to the relevant patient information leaflet.

Firstly, no ‘in‐remission’ participant reported prior knowledge that their foot disease was considered to be ‘in‐remission’, and none remembered this phrase being used by a podiatrist or other healthcare professional during any clinical encounter. Rather than considering themselves to be ‘in‐remission’, a large proportion of participants considered themselves to be ‘healed’.

No…I've never heard it quoted to me at all…we're just told about the ulcers healing

(In‐Remission Participant 08)

Secondly, though many ‘in‐remission’ participants recalled being provided with patient information leaflets during the early stages of their podiatric care, all denied ever having been provided with the ‘in‐remission’ patient information leaflet.

I haven’t had any leaflets regarding that, I’ve had leaflets about looking after your feet, but not ‘in‐remission’

(In‐Remission Participant 05)

Theme 2 Varied perceptions of what the term ‘in‐remission’ means.

People with diabetes classed as in‐remission do not share a common understanding of the term ‘in‐remission’. Sub‐themes associated with participants' varying perceptions of the term include association with cancer care, a perception that ‘in‐remission’ means that foot disease recurrence is inevitable, and a belief in being ‘healed’.

The majority of participants did associate the term ‘in‐remission’ with cancer, but this was not universal. One participant had never previously heard the term ‘in‐remission’ used in any context. Others associated the term with other conditions.

I suppose alcoholism…someone that has maybe been put into a dry‐out clinic, I’d say they would be ‘in‐remission’… and, maybe someone with mental health problems

(In‐Remission Participant 03)

For many, associating the term ‘in‐remission’ with cancer care led to the belief that foot disease recurrence is inevitable and can occur spontaneously.

Does ‘in‐remission’ mean that…it might come back through no interventions that I've done? It just might reappear one day? That's kind of what I think about that phrase

(In‐Remission Participant 04)

The perception that foot disease can recur at any time is rooted in the belief that cancer considered to be ‘in‐remission’ can recur spontaneously. For many, this belief was consolidated by previous lived experience of having had cancer or knowing someone with cancer.

I’ve had cancer too…you don’t have control with the ‘in‐remission’ of cancer

(In‐Remission Participant 02)

Participants also had varying perceptions of the ‘in‐remission’ risk status. Many did not appreciate that they would never regress to ‘low’, ‘moderate’ or even ‘high’ risk. Many believed that they are, or might one day be, ‘healed’.

I think I would say I'm probably healed now

(In‐Remission Participant 05)

Some believed that the absence of active foot disease, regardless of their ‘in‐remission’ risk status, means that the matter is resolved.

I would say, personally, the fact that it managed to clear up is good enough for me

(In‐Remission participant 06)

Theme 3 Highly variable impact of being ‘in‐remission.

Being told that their foot disease is ‘in‐remission’ had a highly variable impact on participants in terms of their engagement with foot care and their mental health.

Participants agreed that being told that they are considered to be ‘in‐remission’ would potentially have a positive effect on their engagement with foot care and that they would be more careful now that they are aware of the high recurrence risk.

If it’s ‘in‐remission’, knowing that there's a chance it could come back again, I would try and do something about it

(In‐Remission participant 06)

Since no participant was previously aware of their ‘in‐remission’ status until participating in this study, this remains a hypothetical outcome of the terminology.

Many participants believed that being ‘in‐remission’ would have a negative psychological impact and cause fear and anxiety.

It's always nagging you as to thinking, well, I'm alright just now…how long is it before it happens again?

(In‐Remission participant 03)

In many cases, this was connected to the perception that disease recurrence was inevitable.

I thought it was healed and gone, that's it, but in‐remission means it's there, latent, and I don't like latent hanging about me

(In‐Remission participant 08)

Additionally, several participants felt that the ‘in‐remission’ status and associated preventative foot care requirements made them a burden on anyone involved in their care.

I would rather it was considered ‘healed’ because I feel as if you're a burden on the NHS, you're a burden on anyone trying to do your feet

(In‐Remission participant 03)

#### 3.1.2 Clinician Themes

Three global themes, each with multiple sub‐themes, were identified in the clinician group summarised in Table 3.

**TABLE 3:** Clinician themes and sub‐themes.

Clinician themes	Sub‐themes
1. Clinician understanding and implementation of the category is inconsistent	1.1 Varied understanding behind rationale for the term ‘in‐remission’
	1.2 Many clinicians do not use the term in discussion with patients
2. Perceptions of the impact on people with diabetes are mixed	2.1 Varied perceptions on how well the term is understood by patients
	2.2 Disagreement among clinicians on how this impacts engagement with foot care and individual engagement is necessary
	2.3 Most clinicians believe ‘in‐remission’ may have a negative psychological impact
3. Risk stratification system facilitates patient education but could be improved	3.1 In‐remission is a useful way to initiate conversation with people with diabetes
	3.2 Patient education should come earlier in the patient journey
	3.3 Current risk stratification is too rigid with the degree of clinical judgement required

Theme 1 Clinician understanding and implementation of the category is inconsistent.

Discussion with clinicians revealed that podiatrists do not share a common understanding of the rationale behind the introduction of the ‘in‐remission’ risk category. Though some clinicians were familiar with the rationale, many were not. Of those who were not aware of the rationale, many perceived the term ‘in‐remission’ to be of more use to clinicians than to people with diabetes.

I don't have a lot of understanding…I think…it's to reflect the significant recurrence rates of diabetic foot ulceration…to potentially make sure that clinicians…are aware of that

(Clinician 12)

As well as an inconsistent understanding of the rationale, clinicians also have a varied approach for implementation of the term ‘in‐remission’ in conversation with patients. Many of the clinicians interviewed do not specifically tell patients with resolved foot disease that they are considered to be ‘in‐remission’.

No, I don’t think I would use the term ‘in‐remission’ to the patient directly

(Clinician 01)

Theme 2 Clinician perceptions on the impact of being classed ‘in‐remission’ on people with diabetes are mixed.

Many clinicians did not believe that the term ‘in‐remission’ is easily understood by the people who are in this risk category. Clinicians disagreed on how being told that they are ‘in‐remission’ impacts a patient's engagement in foot care. Some believe that patients do not attach any significance to this term; others believe that patients misunderstand the concept of ‘remission’ to mean that they are ‘cured’ and can relax their engagement with foot care.

In my opinion, when they hear that they are ‘in‐remission’…they sort of take their foot off the gas for their…concern…they think, ‘ok, that’s me, I’m ‘in‐remission’ now, I can…go back to wearing my unsupportive footwear’

(Clinician 11)

Clinicians also believe that the psychological impact of being told that they are ‘in‐remission’ varies among patients.

There're some patients that would hear that and just don't really care, and then there are other people that'll…I can see that it’s just another thing thrown at them after potentially quite a long, mentally difficult event

(Clinician 12)

Most clinicians agree that any psychological effects that the term ‘in‐remission’ has on their patients would be negative and contribute to a sense of fear and anxiety.

I can see that it may have some really negative and anxiety provoking connotations

(Clinician 03)

There was also agreement among clinicians that being told that they were ‘in‐remission’ would not have any impact on patients with a history of poor engagement.

I think those types of people, I don't think it'll have a massive effect on them because they're not really engaging anyway

(Clinician 01)

Theme 3 Risk stratification system facilitates patient education but could be improved upon.

Clinicians agreed that the current risk stratification tool is a useful facilitator to patient education; however, there was consensus that there is a scope for the current system to be improved. All clinicians agreed that the risk stratification and triage system, particularly with the visual aid of the traffic light system, was a useful means of starting a conversation about the concept of remission and beginning the process of patient education. Clinicians agreed that they would prioritise education over the use of the term ‘in‐remission’ to ensure that the patient had a clear understanding of their risk.

Your risk category now is ‘in‐remission’. Sometimes they think it's over…I think it's just around that education piece afterwards, so they don't run off in their Crocs and live happily ever after.

(Clinician 05)

Clinicians also agreed that education about the risk stratification system should come at an earlier stage in patients' podiatry care rather than waiting until the patient becomes categorised as ‘in‐remission’.

There's a big education piece…around prevention, and I think, with experience, at the beginning of the treatment plan, it's about letting them know what the different risk categories are from low to active so that they know that they'll always be ‘in‐remission’ once they've had… active foot disease

(Clinician 05)

Finally, clinicians agreed that the current risk stratification system is too rigid and that, without an element of subjective clinical judgement, patients can be inappropriately labelled as ‘in‐remission’. This is particularly the case for people with diabetes who sustain a traumatic wound but do not exhibit any other risk factors for foot disease leading clinicians to question whether certain wounds should be documented in this way.

The rigidity of the tool is around that traumatic element. Somebody that is… wearing their flip flops on holiday and they've got no presenting underlying risk factor, but they've got mechanical stress… if they've got a history of diabetes… in the rigidity of this structure, they would be a high‐risk active, high risk in‐remission

(Clinician 08)

## 4 Discussion

We found varied perceptions and implementation of the term ‘in‐remission’ in diabetes foot disease with many of the patients unaware of their risk category and unclear what it means. Similarly, some clinicians were unclear of the background to the introduction of the term and do not use it directly with their patients. Previous research has demonstrated suboptimal levels of lay comprehension of cancer terminology with the term remission one of the least understood [17, 18]. Although people may be familiar with medical terms outside of the clinical setting, this familiarity does not equate to understanding [20]. Furthermore, the educational level was significantly related to the understanding of cancer terminology [18]. Population studies have demonstrated a significant association between multiple deprivation (inclusive of educational attainment) and DFU [21] and higher levels of health literacy are reported as protective for incident ulceration [22]. This may potentially indicate that the misunderstanding of terminology may be more common in a DFU population.

We found consensus among people with diabetes and clinicians that being labelled as ‘in‐remission’ could cause fear and anxiety. The impact of diagnostic labels, such as ‘in‐remission,’ is not well understood, particularly in clinician–patient dialogue and patient facing educational materials. Evidence suggests that such labels are associated with a significant risk of negative psychological consequences [23, 24]. For the people with diabetes, this was often linked to connotations of cancer and the consequent belief that foot disease could recur spontaneously through no fault of their own. Reduced levels of understanding of cancer terminology was linked to increased levels of worry [18], which may further strengthen the perception that the term is not well understood in this context due to the perceived anxiety. Contemporary evidence suggests that messaging, which relies solely on fear, is less effective in achieving behaviour change than strategies which incorporate an element of hope [25]. However, within large complex health systems, the use of diagnostic labels is thought to offer advantages such as supporting access to timely and appropriate treatment and support [24]. The importance of this perceived benefit was noted by several podiatrists who believed the term ‘in‐remission’ raises awareness of a patient's care needs, facilitates triage and allows faster access to podiatry services. However, the recommended actions in the traffic light system are the same for both high‐risk and ‘in‐remission’ categories with only the ‘active’ category requiring different interventions [11].

The people with diabetes in this study were generally unaware of their ‘in‐remission’ status and many podiatrists reported that they did not use the term ‘in‐remission’ directly with patients. Despite the intention of the term to increase patient awareness, podiatrists reported that the lack of use of the term was due to concerns about patient comprehension and potential fear or anxiety. This is contrary to the guidance in the Foot Risk Awareness and Management Education (FRAME) training module currently provided by the NHS in Scotland and England, which states that individuals should be ‘assigned and informed of their risk category’ [26]. All but one of the people with diabetes were unaware of their ‘in‐remission’ risk status and none recalled receiving educational information leaflets that included the term. After being informed of their ‘in‐remission’ status, several patients reported that knowledge of this diagnostic label may potentially increase their engagement with foot self‐care. Further work is required to explore the awareness and impact of foot risk classification status among people with diabetes.

Most clinicians appeared to prioritise education and increasing patients' knowledge over use of the label ‘in‐remission’. This is consistent with the ethos behind the introduction of this term being a starting point to support a conversation around risks of avoidable amputation and death [16]. Current evidence suggests that a single modality of education alone is less effective in preventing ulcer recurrence than complex multi‐faceted interventions [27]. Most clinicians confirmed that they provide patients with relevant information leaflets, but no patient in this study recalled ever having seen the ‘in‐remission’ leaflet. This suggests that written leaflets may not be the most effective means of communicating information about risk stratification. Indeed, verbal education delivered to the patient alone may not be as effective as educational discussions, which involve informal caregivers such as the patient's family or friends [28].

Podiatrists in this study agreed that the risk stratification of people with diabetes requires an element of clinical judgement and that rigid adherence to online systems could lead to inappropriate labelling. This may be pertinent for people with diabetes who do not present with any risk factors but have sustained a break in the skin as a result of trauma. In Scotland, the risk status is automatically calculated by the Scottish Care Information—Diabetes Collaboration system (SCI‐Diabetes) based on the number of risk factors entered into the system. There was no consensus among clinicians as to how a traumatic wound in the absence of any other risk factors should be documented. If a wound is recorded, the resultant ‘in‐remission’ classification cannot be de‐escalated regardless of the number of risk factors present. This resulted in variations in clinical practice reported. The risk stratification process following traumatic wounds in people with diabetes requires clearer guidance.

Alternative terminology was proposed by the clinicians in this study with ‘ulcer‐free’ suggested in preference to ‘in‐remission’. ‘Ulcer‐free’ retains some connotations of cancer care, it specifically connotes the term ‘cancer‐free’. However, the use of the word ‘ulcer’ specifically links this term to foot disease in a way that ‘in‐remission’ does not, emphasising the distinction between diabetes foot disease and cancer. Future work may wish to explore alternative terminology, but this should be carefully developed to ensure psychological impacts are explored prior to implementation. It is recognised in other areas of practice that words can generate positive or negative emotions with resultant impact on behaviour change and potential unintended consequences [29]. Models of communicating the aetiology of DFU to people with diabetes have been proposed and warrant further exploration [30]. The term proposed by the podiatrists in this study (‘ulcer‐free’) could potentially be introduced as a category beyond ‘in‐remission’ after a defined period of time without recurrence of foot disease. As well as providing the potential benefits associated with diagnostic labelling, ‘ulcer‐free is’ positive and provides an element of hope. Furthermore, ‘ulcer‐free’ status could serve as a goal for patients to work towards during periods of remission. Current literature indicates that goal setting has a positive impact on adherence and behaviour change, suggesting that additional goals beyond ‘in‐remission’ may be beneficial in preventing foot disease recurrence [31].

There are several strengths and limitations to this study. To the best of our knowledge, this is the first study to explore perceptions and implementation of the term ‘in‐remission’ in diabetes foot care and important insights have been gained suggesting it may potentially not be working as intended. Further work is required to more fully explore the adoption and perception of this term in diabetes foot care in different populations and taking into account ethnic and cultural considerations. Our sample lacked ethnic and cultural diversity and therefore perhaps has limited relevance beyond the context of people with diabetes and clinicians who are White, British and native English speakers. It must be considered that participants' responses may have been influenced by the context in which they were interviewed and any reluctance to express an opinion could have influenced results and introduced bias [14]. However, all people with diabetes were interviewed individually to facilitate the freedom to express their opinions; topic guides were iteratively developed and all transcripts were returned to all participants for verification to reduce misinterpretation and bias.

## 5 Conclusion

These findings highlight that the awareness, implementation and impact of the term ‘in‐remission’ is inconsistent in podiatry practice. Despite the intention of the term to increase patient awareness, themes were identified regarding applicability and suitability resulting in avoidance by clinicians and confusion and fear from people with diabetes. Although classifying a person with diabetes as ‘in‐remission’ may facilitate triage, access to podiatry services and conversations to support them to avoid amputation and early death; to what extent this is achieved over and above being classed as ‘high‐risk’ is not clear. Further work is required to more fully explore the psychological consequences and impact of the language used in clinical practice and the adoption of terms from one disease to another.

## Author Contributions

**Gordon Donaldson:** conceptualisation (lead), data curation (lead), formal analysis (lead), funding acquisition (lead), investigation (lead), methodology (equal), writing original draft (lead), writing – reviewing and editing (equal). **Gordon Hendry:** conceptualisation (supporting), formal analysis (supporting), funding acquisition (supporting), investigation (supporting), methodology (equal), project administration (supporting), supervision (supporting), writing original draft (supporting), writing – reviewing and editing (equal). **Ruth Barn:** conceptualisation (supporting), formal analysis (supporting), funding acquisition (supporting), investigation (supporting), methodology (equal), project administration (lead), resources (lead), supervision (lead), writing original draft (supporting), writing – reviewing and editing (equal).

## Ethics Statement

Ethical approval was obtained from Glasgow Caledonian University, School of Health and Life Sciences Research Ethics Committee (AHP/A23/026).

## Consent

All participants provided informed consent prior to entry to the study.

## Conflicts of Interest

Gordon Hendry is an Associate Editor for the Journal of Foot and Ankle Research.

## Permission to Reproduce Material From Other Sources

Permission to use Diabetic Foot Risk Stratification and Triage image granted by the Scottish Diabetes Foot Action Group.

## Supporting information

Supporting Information S1

Supporting Information S2

## Data Availability

Data are available upon request.
