# Supplementary material for: Exploring Patient and Podiatrist Perspectives of the ‘In‐Remission’ Status in Diabetes‐Related Foot Disease
Source: J Foot Ankle Res. 2025 May 13;18(2):e70045. doi: 10.1002/jfa2.70045 (PMC12069974; doi:10.1002/jfa2.70045)
Supplement: Supplementary file 2 — Supporting Information S2 [file JFA2-18-e70045-s002.docx]

Appendix 2: Topic Guide (Clinicians)

**Exploring Patient and Clinician Perceptions of the term ‘In Remission’ in the Context of Diabetic Foot Ulceration**

Researcher Prompts:

- Introduction
- Overview of topic and rationale for interview
- Establish that there are no right or wrong answers
- Approximate duration
- Reminder of anonymity, confidentiality, and ability to withdraw at any time
- Reminder that conversations will be audio recorded
- Confirm understanding and allow for questions and clarifications
- Confirm consent
- Begin by giving rundown of structure: three main parts to the discussion, going to begin by learning more about your background, move on to discuss your opinions on the term ‘in remission’, then going to talk more about how it might impact your patients’ engagement in personal foot care

**Part One: Background**

- 1. How long have you been in professional practice?
  2. What proportion of your clinical time is spent working with people with diabetes?
  3. Do you often see patients during periods of remission, or do you more commonly see patients during periods of active foot disease?

**Part Two: Understanding and Interpretation of ‘In Remission’**

- 1. What do you know about the term remission in healthcare/medicine?
  2. Can you tell me about your knowledge of the use of the term in remission in diabetes foot disease?
  3. How do you feel about the use of the term ‘in remission’ in relation to foot disease in diabetes?
  4. What is your understanding of the ‘in remission’ risk status in relation to its intended impact?
- the rationale behind it?
  1. What connotations do you think the term ‘in remission’ might have?
- Positive or negative?
- Fear-based or empowering?
  1. Can you tell me if and how you use the term ‘in remission’ in discussion with your patients who are in this category?
  2. How easily understood do you think this term is for your patients?
  3. Do your patients ever comment on this term or ask further questions about it?
  4. Have you ever communicated this information to a patient via an interpreter or used the term with someone who speaks english as a 2^nd^ language?
- made use of the translations of the ‘In Remission’ patient information leaflet?
- If so, did the patient require any further clarification?

**Part Three: Impact of ‘In Remission’ on Personal Foot Care**

- 1. Do you think that being ‘in remission’ impacts how your patients care for their feet?
- Skin care, nail care, footwear, foot inspection, podiatry appointments
  1. Do you think being ‘in remission’ may have a psychological impact on your patients?
- Do you think that might impact their engagement in personal foot care?
  1. Do you have any suggestions for alternative terms or messaging that may help patients care for their feet?

Topic Guide (Patients)

**Exploring Patient and Clinician Perceptions of the term ‘In Remission’ in the Context of Diabetic Foot Ulceration**

Researcher Prompts:

- Introduction
- Overview of topic and rationale for interview
- Establish that there are no right or wrong answers and some answers may take a while to think about so don’t worry about time
- Approximate duration
- Reminder of anonymity, confidentiality, and ability to withdraw at any time
- Reminder that conversations will be audio recorded
- Confirm understanding and allow for questions and clarifications
- Confirm consent
- Begin by giving rundown of structure: two main parts to the discussion, going to begin by finding out more about what being in remission means to you, then going to talk more about how being in remission affects how you care for your feet

**Part One: Background**

1. What type of diabetes do you have? (type 1 or type 2)
2. How long have you had diabetes?
3. How many times have you had a foot ulcer (foot wound) due to your diabetes?
4. Is English your first language?
   1. If no, what is your first language?
5. How often do you need to have someone help you when you read instructions, pamphlets, or other written material from your doctor or pharmacy?

**Part Two: Understanding and Interpretation of ‘In Remission’**

- 1. What do you know about the term ‘in-remission’ in medicine/healthcare?
  - Have you heard the term before? Where?
  - What does it make you think of?
  1. What about the use in remission in diabetes? Are you aware that it is used in diabetes?
- Have you seen any of the ‘in remission’ leaflets? (show them leaflet if on video)
- Have you accessed these in any language other than english? What are the connotations of this word in your language?
  1. Are you aware that your foot ulcer is currently considered to be ‘in remission’?
- How does this make you feel?
  - Does it make sense to you?
  - Do you feel you understand what it means?
  - Can you try to explain it in your own words?
  - Does it feel relevant? Meaningful?
  - *Provide definition if patient unclear*
  1. How do you feel about your foot problem being considered as ‘in remission’ rather than healed?
- How has this affected how you feel about your risk of a foot ulcer?
- Do you understand why your healthcare team would use that term
  - *Offer summary that data shows recurrence is likely if patient unclear*
  1. Do you feel that someone who is ‘in remission’ is at greater risk or less at risk of ulceration than someone who is considered to be ‘high risk’?

**Part Three: Impact of ‘In Remission’ on Personal Foot Care**

- 1. How do you look after your feet?
  2. Has your foot care routine changed as a result of being ‘in remission’?
- To what extent has the level of risk associated with being ‘in remission’ impacted your personal foot care?
  1. Does being ‘in remission’ have a psychological impact?
- How did this risk status make you feel?
  1. Do you have any suggestions for alternative terms or messaging that may help you care for their feet?

**That brings us to the end of the interview, thank you again for your time it’s much appreciated**
